# P-1042. Multimodal Intervention Combining Education, Checklists, and Feedback to Sustain Low CLABSI Rates

**DOI:** 10.1093/ofid/ofaf695.1237

**Published:** 2026-01-11

**Authors:** Leena James, Jomel Raju

**Affiliations:** St. Joseph's College of Pharmacy, Elampally, Kerala, India; St. Joseph's College of Pharmacy, Cherthala, Pala, Kerala, India

## Abstract

**Background:**

Achieving and sustaining low Central Line-Associated Bloodstream Infection (CLABSI) rates is a major challenge in resource-limited healthcare settings. This study evaluated the impact of a multimodal intervention incorporating education, standardized checklists, and structured feedback mechanisms to maintain low CLABSI rates over a 2-year period in a rural tertiary care hospital ICU in South India.

**Figure:**
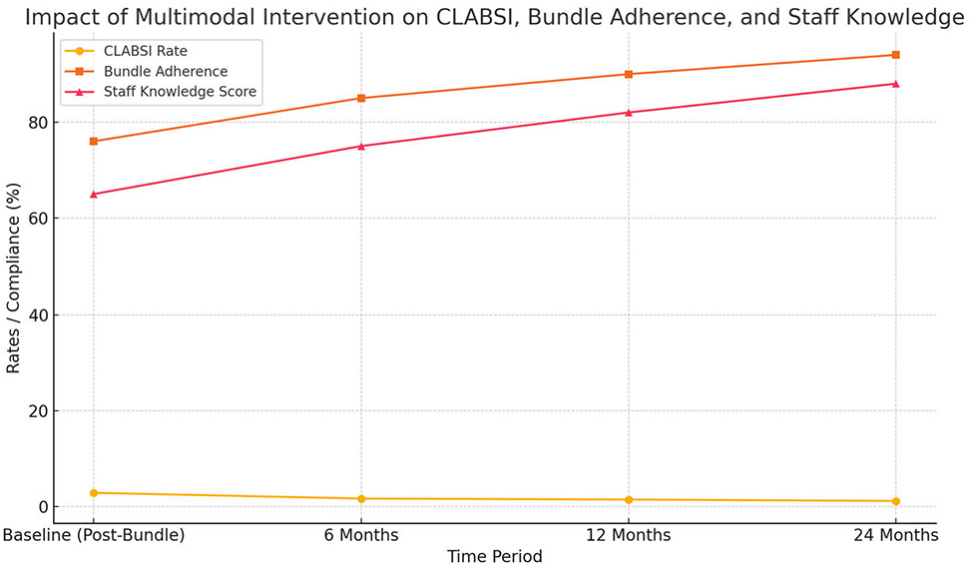
Impact of Multimodal Intervention on CLABSI Rates, Bundle Adherence, and Staff Knowledge Over 24 Months This graph illustrates the outcomes of implementing a multimodal strategy incorporating education, checklist audits, and structured feedback in a 12-bed adult ICU of a tertiary rural hospital in South India. Over a 24-month period (2023–2024), Central Line-Associated Bloodstream Infection (CLABSI) rates decreased from 2.9 to 1.2 per 1000 catheter-days, while bundle adherence improved from 76% to 94%, and staff knowledge scores rose from 65% to 88%. Sustained improvements demonstrate the effectiveness of continuous education, monitoring, and feedback in maintaining infection control gains in resource-limited settings.

**Methods:**

A prospective interventional study was conducted in the 12-bed adult ICU of a tertiary-level hospital in rural South India from January 2023 to December 2024. Following a prior successful bundle implementation phase (2022), the multimodal intervention included:

(1) Monthly educational workshops focused on central line insertion and maintenance practices,

(2) Daily completion of central line maintenance checklists, supervised by nurse leaders,

(3) Monthly feedback sessions reporting compliance rates and infection data to clinical teams.

CLABSI rates were monitored monthly (per 1000 catheter-days) and compared using interrupted time series (ITS) and chi-square analysis.

**Results:**

The baseline CLABSI rate post-bundle phase was 2.9 per 1000 catheter-days. After multimodal implementation, rates were maintained at 1.7, 1.5, and 1.2 per 1000 catheter-days at 6, 12, and 24 months respectively (p < 0.001). Bundle adherence improved from 76% to 94% (p < 0.001), and staff knowledge scores increased from 65% to 88% (p < 0.001). An estimated 24 infections were prevented, corresponding to approximately INR 13.6 lakh (∼USD 16,200) in healthcare cost savings.

**Conclusion:**

A multimodal strategy combining education, checklist-based monitoring, and real-time feedback was highly effective in sustaining low CLABSI rates in a resource-limited, rural tertiary ICU. Engaging frontline staff through continuous education and performance feedback was critical to long-term success.

**Disclosures:**

All Authors: No reported disclosures

